# Concreteness ratings for 36,000 Estonian words

**DOI:** 10.3758/s13428-023-02257-4

**Published:** 2023-12-21

**Authors:** Mariann Proos, Mari Aigro

**Affiliations:** 1 University of Tartu, Jakobi 2-446, Tartu, Estonia; 2 University of Tartu, Jakobi 2-430, Tartu, Estonia

**Keywords:** Estonian, Concreteness, Semantic ratings

## Abstract

We present a collection of concreteness ratings for 35,979 words in Estonian. The data were collected via a web application from 2278 native Estonian speakers. Human ratings of concreteness have not been collected for Estonian beforehand. We compare our results to Aedmaa et al. (2018), who assigned concreteness ratings to 240,000 Estonian words by means of machine learning. We show that while these two datasets show reasonable correlation (*R* = 0.71), there are considerable differences in the distribution of the ratings, which we discuss in this paper. Furthermore, the results also raise questions about the importance of the type of scale used for collecting ratings. While most other datasets have been compiled based on questionnaires entailing five- or seven-point Likert scales, we used a continuous 0–10 scale. Comparing our rating distribution to those of other studies, we found that it is most similar to the distribution in Lahl et al. (*Behavior Research Methods,* *41*(1), 13–19, 2009), who also used a 0–10 scale. Concreteness ratings for Estonian words are available at OSF.

## Introduction

Concreteness ratings are a valuable resource, and are utilized in many different fields, including linguistics, psychology, language technology, and marketing research. Muraki, Abdalla, Brysbaert, and Pexman (2022) have provided a thorough overview of the wide range of applications for concreteness ratings. The linguistic research problems that may be addressed with concreteness ratings include the effect of concreteness in mental grammar and language processing (Papitto, Lugli, Borghi, Pellicano, & Binskofski, 2021; Li et al., 2021), its role in various neurological disorders (Smirnova, Clark, Jablensky, & Badcock, 2017; Alyahya, Halai, Conroy, & Lambon Ralph, 2018; Benau et al., 2019), and the storage and retrieval of lexical items in memory (Chubala, Surprenant, Neath, & Quinlan, 2018; Tse & Altarriba, 2022). However, the nature of concreteness as a cognitive-semantic dimension is still debated.

Concreteness and abstractness are semantic characteristics of concepts, and all concepts (represented by words) may be viewed as situated somewhere on a concreteness–abstractness continuum. There are several theories as to how concreteness and abstractness relate to the way we understand and process concepts. One of the more predominant theories is the dual-coding theory from (Paivio, 1971, 1991). The theory posits that all concepts are coded on the verbal level, but concrete concepts, unlike abstract concepts, are coded on both the verbal and the sensomotoric level. Hence, concrete concepts are coded by two distinct means, making them easier to retrieve than abstract concepts, which only rely on verbal associations. Experiments have indeed shown that participants conduct various memory-related tasks faster with concrete concepts, compared to abstract concepts, offering support to some type of divergence in their processing patterns (Paivio, 1965; Paivio, Yuille, & Madigan, 1968).

More recently, the majority of work on the concreteness of concepts has concentrated on their grounded nature. More specifically, the grounded theory suggests that all concepts are grounded in perceptual experience (Barsalou, 1999; Barsalou, Kyle Simmons, Barbey, & Wilson, 2003; Barsalou, 2008) and that the distinction between abstract and concrete concepts might not be as binary as researchers have thought (Barsalou, 2020). In this framework, concrete and abstract concepts are viewed as being grounded in distinct modalities. For example, concrete concepts tend to be rated higher in haptic, visual, and olfactory modalities, while abstract concepts are strongly associated with auditory and introspective modalities (Connell, Lynott, & Banks, 2018). Abstract concepts have also been found to be grounded in social and linguistic experiences (Borghi, 2020). fMRI evidence has shown highly abstract concepts such as science terminology could be grounded in experiential information (Ulrich, Harpaintner, Trumpp, Berger, & Kiefer, 2022).

However, other evidence shows that abstract concepts may not form a homogeneous group as they could be grounded in distinct experiences and cognitive faculties. Troche, Crutch, and Reilly (2014, 2017) use various cognitive dimensions to characterise the semantic space of concepts, creating a multimodal space where concepts vary along a number of continua. Similarly, Villani, Lugli, Liuzza, and Borghi (2019) use a variety of cognitive dimensions indicating four distinct types of abstract concepts. Distinct neural circuits can be observed to be active during the processing of different sets of semantically predefined abstract concepts (Kiefer, Pielke, & Trumpp, 2022), and different types of abstract verbs rely on distinct underlying representations (Muraki, Cortese, Protzner, & Pexman, 2020). In a systematic review of 60 studies investigating abstract concepts, Conca, Borsa, Cappa, and Catricalá (2021) show consistent evidence for the heterogeneity of abstract concepts from behavioral, clinical, and neuroimaging studies.

In any case, most agree that concreteness constitutes a property with profound consequences for the processing of (linguistic) concepts and their mental representation. In light of this, the past few decades have seen the creation of concreteness rating collections in a large number of languages. Most collections reflect human ratings where judgements have been collected and averaged across a number of participants. For most languages, these databases range between 1,000 and 4,000 words, e.g., Chinese (Liu, Shu, & Li, 2007), German (Lahl, Göritz, Pietrowsky, & Rosenberg, 2009), Spanish (Guasch, Ferré, & Fraga, 2016), Indonesian (Sianipar, van Gronestijn, & Dijkstra, 2016), Portuguese (Soares, Costa, Machado, Comesañ, & Olieveira, 2017), French (Bonin, Méot, & Bugaiska, 2018) and Croatian (Ćoso, Guasch, Ferré, & Hinojosa, 2019). These studies have generally collected concreteness ratings alongside other psycholinguistic ratings, including effectiveness, imageability, and age of acquisition. For a few languages, however, concreteness index databases are extensive. For instance, the English collection includes 40,000 words (Brysbaert, Warriner, & Kuperman, 2014b) and 62,000 multiword expressions (Muraki et al., 2022). In addition, Brysbaert, Stevens, De Deyne, Voorspoels, and Storms (2014a) have collected ratings for 30,000 Dutch words, and Xu and Li (2020) for 10,000 two-character words in Mandarin Chinese.

Interestingly, there does not seem to be one universal distribution pattern for these ratings. Some studies show a binomial tendency in the distribution with extreme values being more represented than those somewhere in the middle (e.g., Imbir, 2016; Xu & Li, 2020). Others show bias toward the abstract anchor point (e.g., Brysbaert et al. 2014a; Sianipar et al. 2016) while others are skewed toward the concrete anchor point (e.g., Lahl et al. 2009). In terms of participant agreement, most studies find that the correlation coefficient between different sets of human ratings for the same words to be around 0.8–0.9. For instance, correlation of 0.91 was shown in Brysbaert et al. (2014b), 0.88 in Guasch et al. (2016), 0.84 in Lahl et al. (2009) and 0.73 in Ćoso et al. (2019).

Another interesting topic emerging from recent concreteness studies has to do with the effect different collection methods have on rating values. Collecting human ratings is costly in terms of time and resources, especially for languages with limited online crowd sourcing options. A number of studies have therefore used machine learning to assign various semantic ratings to a large amount of words. They include both valance and arousal ratings (Palogiannidi, Iosif, Koutsakis, & Potamianos, 2015; Sedoc, Preoţiuc-Pietro, & Ungar, 2017; Vankrunkelsven, Verheyen, Storms, & De Deyne, 2018), as well as concreteness ratings (Vankrunkelsven et al., 2018; Aedmaa, Köper, & Schulte im Walde, 2018; Ivanov & Solovyev, 2022).

One such study is that of Aedmaa et al. (2018) who followed the procedure in Köper and Schulte imWalde (2016). First, the nearly 30,000 English words with human ratings in Brysbaert et al. (2014b) were automatically translated into Estonian. Words from a 170-million token corpus were then used to create a vector space model describing the lexical composition of the context in which each word occurs. Machine translated words were used as seeds for an algorithm (Turney & Littman, 2003) that used them to learn to assign concreteness ratings to these words, based on the assumption that the concreteness of a word’s context is informative of the lexical concreteness of that word. This resulted in a list of 240,000 Estonian words with machine learning-based concreteness ratings.

Previous studies on comparison between human and machine ratings have shown that distributional semantics methods in machine learning can mirror human behavior as they report correlation with human ratings similar to that between human-to-human correlation rates. For example, Köper and Schulte imWalde (2016) assign machine learning based ratings of abstractness, arousal, imageability, and valence to 350,000 German words, report a correlation coefficient of 0.8 between human ratings, and machine ratings (most human ratings originating from English word variants). Vankrunkelsven et al. (2018) report a correlation of 0.87 between machine ratings and more than 30,000 human concreteness ratings from Brysbaert et al. (2014b). In this perspective, the achieved correlation between human and machine-based ratings appears to validate the method as a productive predictor of words’ semantic properties.

Estonian presents us with an interesting opportunity. In addition to investigating the concreteness of Estonian words and producing a highly useful dataset, we can also compare human ratings to an existing machine learning- based ratings dataset compiled by Aedmaa et al. (2018), described above.

In this paper, we present the first concreteness indexes for Estonian words (*n* = 35,979) collected from humans. We discuss their overall distribution and participant agreement. These data are compared to machine learning based Estonian word ratings (Aedmaa et al., 2018), discussing the main distinctions and their potential source.

## Method

We designed a lexical judgement task to collect concreteness ratings for 40,000 words. We used a combination of three different sources for the list of words included: the Estonian Reference Corpus (2018), the frequency table of the 1000 most frequent words in the Phonetic Corpus of Estonian Spontaneous Speech (Lippus, 2019), and the Basic Estonian Dictionary (Tuulik, Tiits, Kallas, Koppel, & Jürviste, 2014), including 5,000 most essential lexemes for L2 learners. On the one hand, we aimed to create a list of *frequent* lexical items, which is why we used written (Estonian Reference Corpus) and spoken (Phonetic Corpus of Estonian Spontaneous Speech) corpus frequency lists. On the other hand, the list was also intended to reflect common items, which is why items were added from the Basic Estonian Dictionary. The following section describes the compilation of the word list and the procedure of the experiment.

### Material

First, we extracted the 40,000 most frequent words (nouns, adjectives, verbs, adverbs, pronouns and adpositions) from the Estonian Reference Corpus—a 242 million token text collection representing written Estonian, mostly journalism texts. Next, items among the 1,000 most frequent words in the Phonetic Corpus of Estonian Spontaneous Speech (Lippus, 2019) that were missing from that list were added, pushing out the least frequent items to keep the total at 40,000. This corpus is a collection of spontaneous speech transcriptions including approx. 700,000 tokens. Items from the Basic Estonian Dictionary were added in the same manner.

As the last step in list creation, we compiled a list of 111 homonymous words, partially based on the Handbook of Estonian (Ross, Erelt, & Erelt, 2007), and partially by manually checking the existing list. These are lexemes, the reference forms (nominative singular, infinitive) of which have several unrelated meanings, e.g., *aas* ‘meadow’/‘loop’. These lexemes were divided into several items in the main list, reflecting their distinct meanings.^1^ Each copy of the lexeme was given a short description about the meaning that we intended the judgement to reflect, which was presented to the participant together with that word. These descriptions belonged to one of two types. In most instances, participants were merely given the nominative, genitive, and partitive forms of homophonous words as the two meanings had distinct inflections in other cells. For example, one instance of *mure* was accompanied by *mure, mure, mure*, referring to the meaning ‘worry’. Another was accompanied by *mure, mureda, muredat*, indicating the meaning ‘crumbly’. However, for a minority of homonyms with identical case forms, minimal sentence context was presented. For instance, one instance of *puur* was accompanied by *Lind on puuris.* (‘The bird is in the cage.’) indicating ‘cage’ meaning, while the other was accompanied by *Töömehel on mitu puuri.* ‘The handyman has multiple drills’, indicating ‘drill’ meaning. The dataset of homonymous lexemes in Estonian is freely available (Aigro, 2022).

### Procedure

Participants saw the experiment in a web application, the link of which was advertised in online channels and shared on social media. Participants were first presented with task description, where abstractness and concreteness were explained by text summarized in the following^2^: *In this task, we want to find out more about concreteness and abstractness. Some words are concrete, because they are very strongly connected to our five senses (seeing, hearing, smelling, tasting, feeling) — ‘pen’ is one such word. Other words are considered abstract, because it is very difficult to sense them with our physical senses, but the concepts exist nonetheless — ‘friendship’ is one such word. There are also some words that are somewhere in between — you might be able to experience these through your senses to a degree, but to understand these words you also rely on language*.

After reading the instructions, participants ticked a box to give their informed consent, also confirming they were older than 18 years and native Estonian speakers. They then filled out a questionnaire collecting metadata.

The participants moved on to the practice phase, where they were introduced to the layout of the rating task. In this phase they rated ten words. The ten practice phase items included verbs, nouns and adverbs. For two words, syntactic category was highlighted (noun, adjective) to introduce participants to the variety of words they would see during the task. One practice phase word was a homonym with accompanying text. Five items were designed to make reference to distinct senses. They included *hais* ‘stench’ for smell, *vilistama* ‘whistle’ for sound, *suhkur* ‘sugar’ for taste, *näpuvigastus* ‘finger injury’ for touch and *kuusk* ‘spruce’ for sight. In order to move on to the next item, participants had to move the rating indicator on a scale beneath the word, thus assigning each with a concreteness value. Participants were instructed to tick the ‘I do not know this word’ box if they were not familiar with the meaning of the presented word.

Instead of the more common Likert scale, we used an entirely gradient scale. Participants chose a rating between 0 and 10 by dragging an indicator on an axis, placing it in the desired place. The scale was a straight line with ‘Very abstract’ written in the far left end and ‘Very concrete’ written in the far right end. Eleven integer numbers were visible with equal intervals below the scale, but the participant was able to drag the indicator to any point on the scale, including non-integer numbers. The gradient scale was chosen to allow for a more symmetrical representation of the scale, and for the comparison with Aedmaa et al. (2018) to be more accurate.

During the experiment, each participant was presented with 215 stimuli, which included 200 random words from the total list of 40,000 words, ten calibrator words and five control words. Calibrator and control words were identical for all participants. The calibrator words included five items expected to receive a high concreteness rating (e.g., *põrsas* ‘piglet’), as well as five items we expected to be highly abstract (e.g., *headus* ‘goodness’). Their purpose was to determine if participants interpreted the scale in the intended manner (with 10 signifying high and 0 low concreteness). Control words, i.e., nonce words with no meaning in Estonian (e.g., *pooner*), were used to filter out participants who did not tick the ‘I do not know this word’ box and therefore provided unreliable data.

Participants were recruited by way of mailing lists, social media (including paid social media advertisements), and physical advertisements. Experiment design assumed a minimum of 2,000 participants for each item to receive ten judgements. For their effort, participants were entered in a raffle for a gift card with a 10% chance of winning. Data collection lasted from January 2021 to October 2022.

## Results

### Data trimming

During the testing period, a total of 472,216 ratings were collected from 3,424 participants. We used a number of criteria to exclude the data of the least reliable raters. All ratings of a participant were removed if at least one of the following conditions applied:

- the participant completed less than 25% of the task;
- the participant did not meet the age requirement as evident from the questionnaire;
- the participant gave the same rating to 30 or more consecutive items;
- the participant gave a rating to 3 or more nonce words included for control.

These criteria resulted in the exclusion of all ratings by 1,146 (33.5% of) participants (*n* = 56,666, 12% of ratings). Most participants (84% of the 1,146 excluded participants) were excluded due to completing less than 25% of the task.

In addition, lexical items, the meaning of which was unknown to three or more participants, were removed from the dataset (*n* = 334). Such words were judged to be unlikely to be known well enough for the existing ratings to be informative (see also Brysbaert, Warriner, & Kuperman, 2014b).

Furthermore, the final dataset only reports on ratings for 35,979 words, i.e., words with nine or more ratings. Admittedly, this number is on the low side, e.g., Brysbaert et al. (2014b) report aiming for 30 ratings per word but some words achieving less than 20 in the end. However, aiming to achieve 30 ratings per word for nearly 40,000 words as in Brysbaert et al. (2014b) is unfeasible for Estonian for several reasons. First, there are no crowdsourcing tools for such a study (e.g., Mechanical Turk), meaning researchers must market and advertise the study via mailing lists and social media. Second, there are approximately 900,000 native Estonian speakers in the country, meaning that a stark increase in participant number goal would make the task exponentially more laborious. The study already includes approximately 0.4% of all Estonian speakers in the country.

**Fig. 1:**
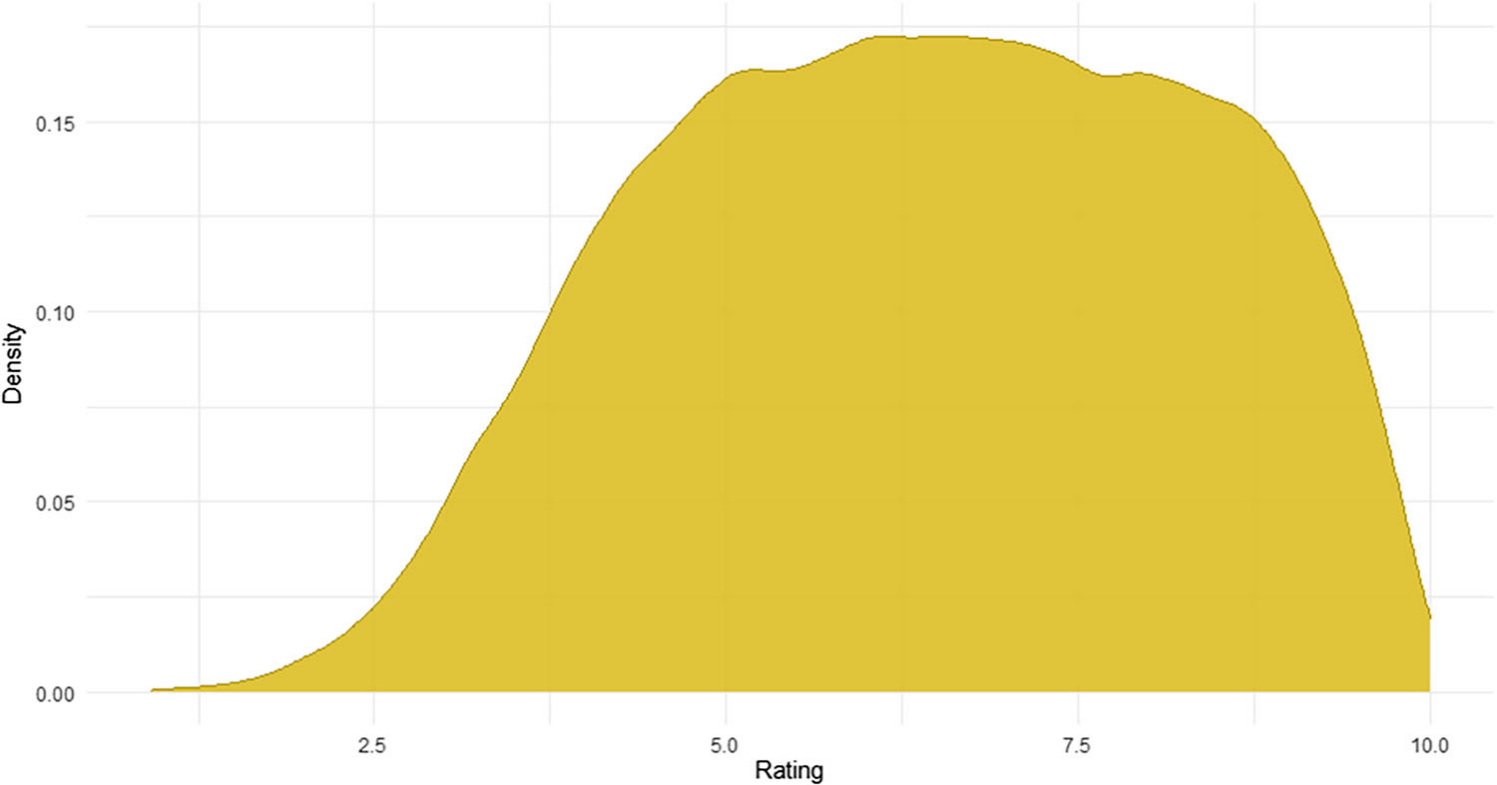
Density plot of concreteness ratings for 35,979 Estonian words (bandwidth method: Sheather and Jones)

**Fig. 2:**
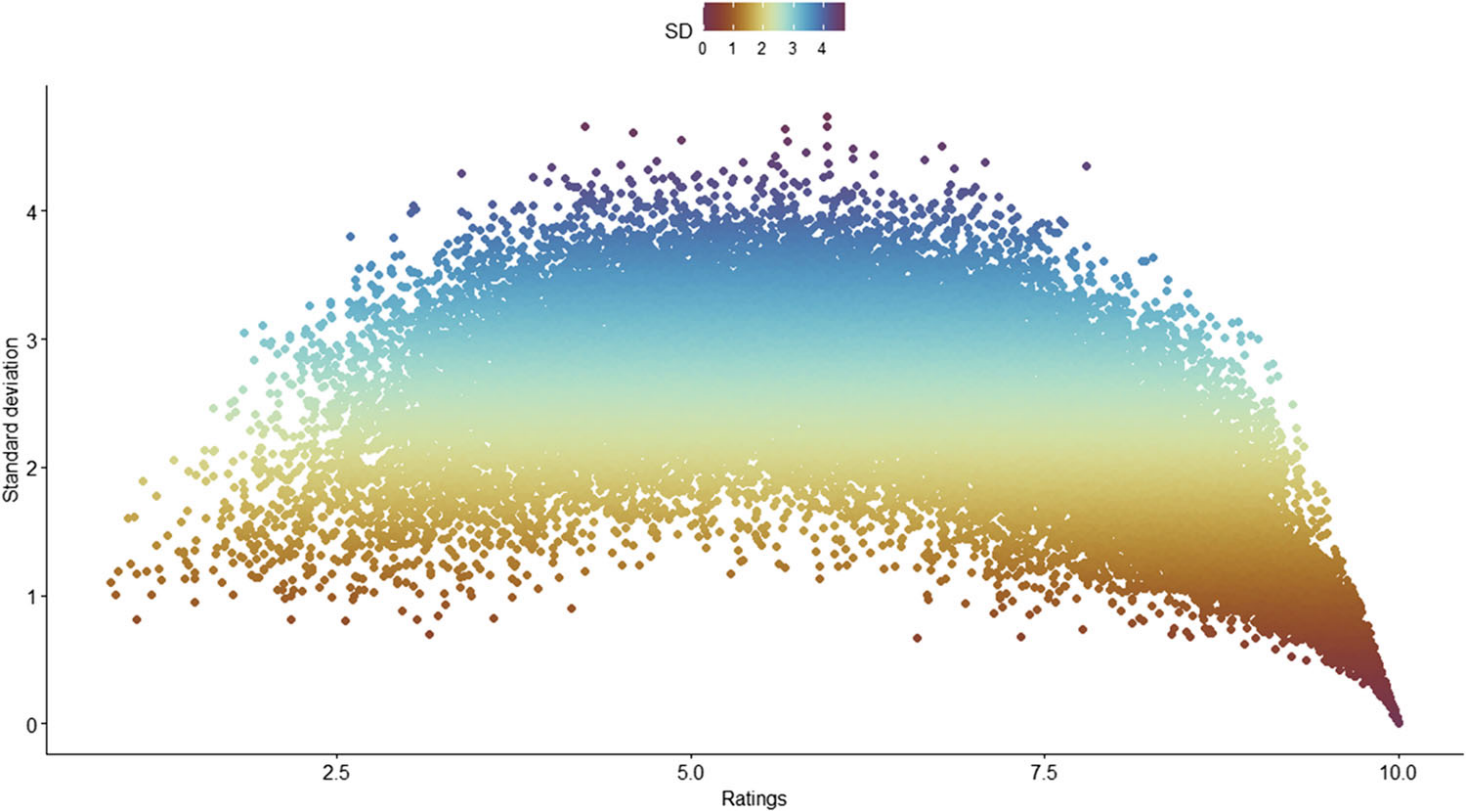
Scatterplot of mean ratings and their corresponding standard deviations

Altogether, 17.4% of collected data was omitted from the final dataset; 11.5% is due to removing participants or individual words with unreliable ratings. Another 5.9% is made up of ratings for words with less than nine ratings overall. Such exclusion rates are to be expected, considering that the web-based study was not published on a research participant platform, but was instead marketed on social media, attracting people much less likely to finish the task. Hence, when researching a language with a limited pool of native speakers, more data will likely be eliminated than in studies focusing on *lingua francas*. Similar studies include Ćoso et al. (2019) who report excluding 11% of ratings and Lahl et al. (2009) who report 18%: comparable to the present study.

**Fig. 3:**
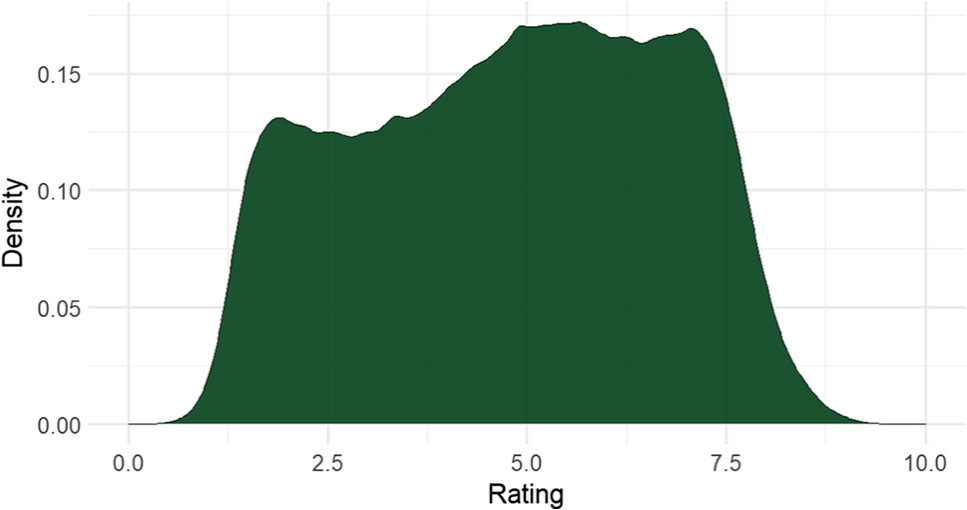
Density plot of concreteness ratings for 243,674 words from Aedmaa et al. (2018) (bandwidth method: Sheather and Jones)

**Fig. 4:**
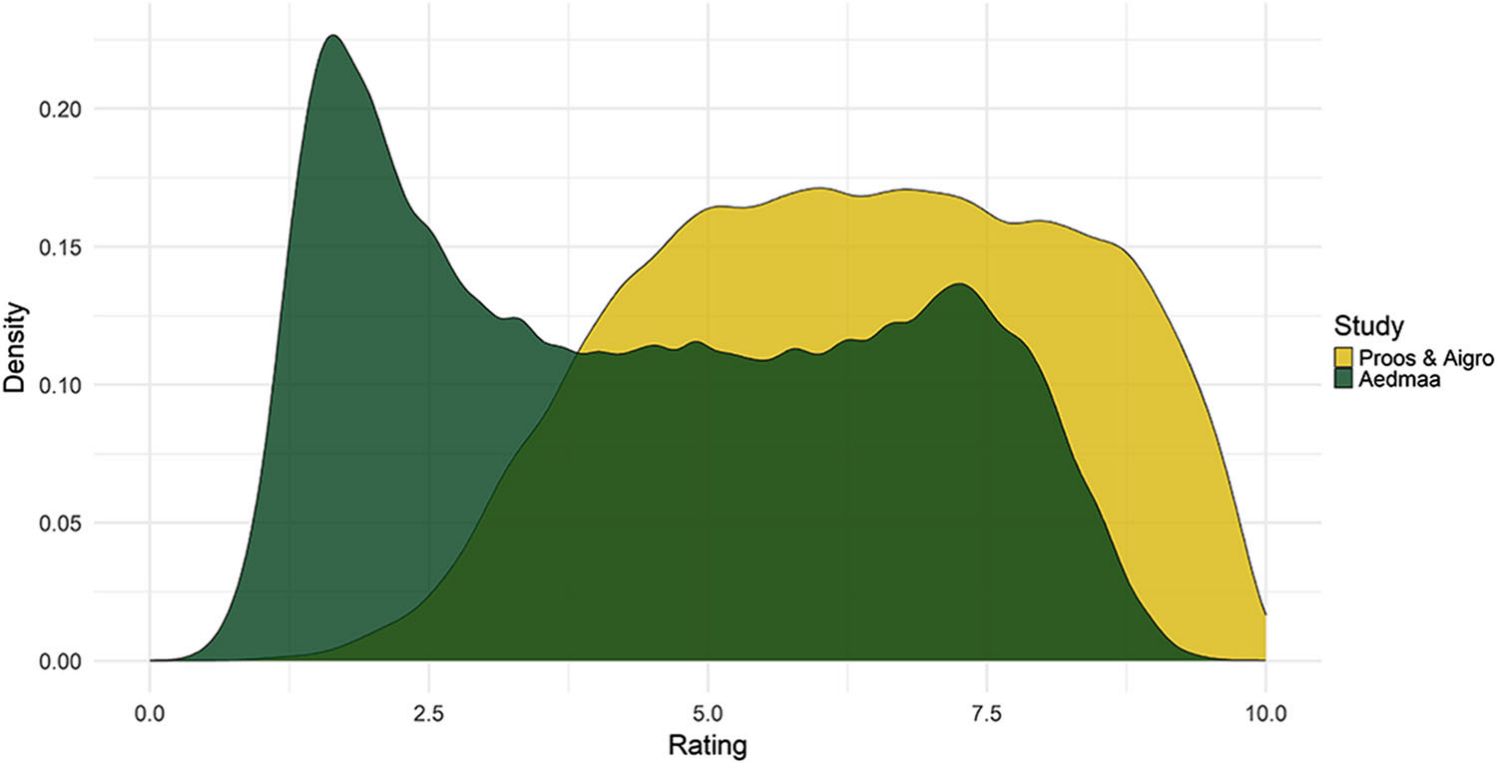
Density plot of ratings (35,117 overlapping words) from Aedmaa et al. (2018) (*green*) and the present study (bandwidth method: Sheather and Jones)

The final dataset includes data from 2,278 participants (mean ratings per word = 10.8, mean ratings per participant = 171) and a total of 389,658 ratings for 35,992 words. They include 1,992 women (87%), 268 men and 18 selected other for their gender (mean age = 44.3, SD = 14.7). Out of the 2,278 participants, 1,585 had a university degree.

### Overview of collected ratings

The distribution of ratings is presented in Fig. 1 via a density plot, based on kernel estimates. The distribution of ratings is skewed towards the concrete side of the scale as an especially high proportion of words have a mean rating between 5 and 9. There is no sign of bimodality in the ratings.

Figure 2 presents ratings together with their respective standard deviations (SD, mean = 2.4). Lower SD values indicating higher agreement rate between participants are found in the higher end of the concreteness scale, i.e., for highly concrete concepts. There is a large concentration of near-zero SDs for words with an average concreteness rating of 10, meaning people largely agree on the rating of very concrete concepts. This also applies to the lower end of the concreteness scale to some extent, but the effect there is much weaker. The highest SDs indicative of little agreement are observed in the middle part of the scale. We did not exclude any ratings on the basis of their standard deviation.

### Comparison with machine-created ratings

As discussed in “Introduction” section, Aedmaa et al. (2018) assigned concreteness ratings to more than 240,000 Estonian words by means of semantic vectors. We plotted the distribution of the ratings from Aedmaa et al. (2018) in Fig. :

Figure presents the distribution of all ratings in Aedmaa et al. (2018) while Fig. 4 only shows the words which also occur in our study (*n* = 35,117). Figure shows slight bimodality with a higher proportion of ratings in the concrete end of the scale. When only looking at ratings overlapping with the present study (Fig. 4), bimodality is stronger and most lexemes have a more abstract rating. No bimodality, however, is seen in human ratings, which lean more heavily on the concrete side.

**Fig. 5:**
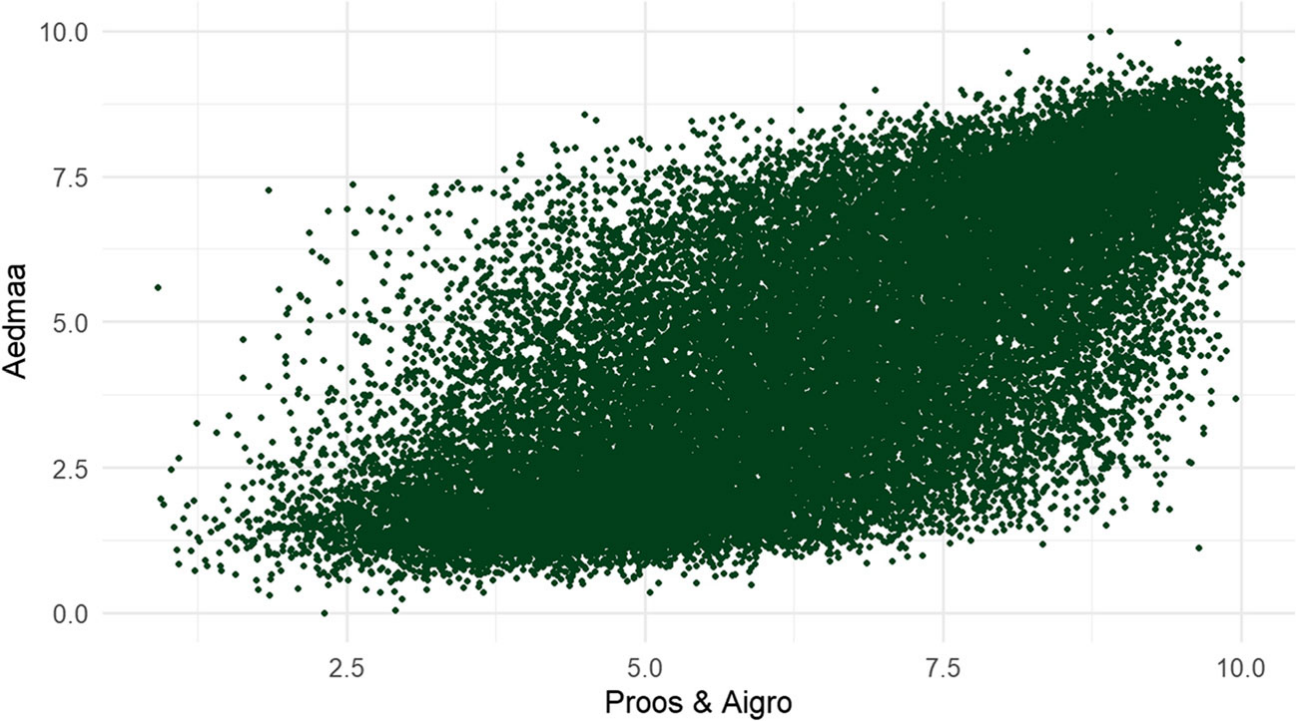
Scatterplot of Aedmaa et al. (2018) ratings and ratings from the present study (*R* = 0.71, $\textit{p} < 2,2e-16$)

Following Köper and Schulte imWalde (2016), we correlated our human ratings with ratings from Aedmaa et al. (2018) by applying a linear regression model. Figure 5 illustrates the correlation between the machine ratings from Aedmaa et al. (2018), and the human ratings presented in this study:

The correlation between the two sets of ratings (*R* = 0.71) is broadly similar to but slightly lower than other reported correlation values (see “Introduction” section). However, there is a notable difference in the distribution of the two sets of ratings, as seen on Fig. 4. Ratings in Aedmaa et al. (2018) have a high peak around the 1–2 region of the scale, while human ratings in that region are rather rare. As is evident from the scatterplot, both the high abstractness word set as well as the high concreteness word set in Aedmaa et al. (2018) receive more varied ratings when judged by human raters, leading them to be more widely distributed across the scale.

To further illustrate the differences between machine ratings and human ratings, Table 1 presents the top words with largest negative residuals in the regression model, i.e., words that were rated to be significantly more abstract by humans.

Almost all words in Table 1 are compounds, and more importantly, quite a few of them are *ad hoc* compounds with very specific, context-requiring semantics. For example, it is unclear without context what could be the referent of a ‘spring circle’. While the machine ratings for these compounds lean more towards the concrete end of the scale, humans rated these to be highly abstract. This is likely because these lexemes were likely used metaphorically inside a highly concrete context, which was available for the algorithm (e.g., *pallivõlur* ‘ball wizard’ occurring in a sports text describing a highly physical event. Without context, human raters regard such *ad hoc* compounds as vague novel concepts, judging them as abstract to reflect this semantic vagueness. This highlights the role of context-dependability as a lexical variable, which affects concreteness values differently in distinct experiment designs.

To check whether there could be a systematic difference between how compounds are rated by humans vs. the algorithm, we extracted all compounds in the overlapping dataset between the present study and Aedmaa et al. (2018) (*n* = 18,673). For compounds, Pearson’s correlation between the two ratings is 0.66, which is statistically significantly lower (Fisher’s *Z* = -10.4189, $\textit{p} < 0.001$)^3^ than for the entire dataset (*R* = 0.71).

This result also highlights what can be considered a shortcoming of our list. The list includes words that are not in common use, mainly because of the nature of the corpus we used to compile the list. Since the corpus is relatively small compared to newer ones, some words become over-represented, thus they are quite frequent in the corpus, but not very frequent in everyday language use.

**Table 1:** Top ten words with largest negative model residuals, i.e., words which were rated to be significantly more abstract in the present study than in Aedmaa et al. (2018)

word	Translation	Proos & Aigro	Aedmaa	Predicted	Residual
*täismõõde*	full dimension	1.847	7.267	8.030	-6.183
*ajahammas*	tooth of time	0.912	5.590	7.053	-6.141
*särasilm*	bright-eye	2.552	7.358	8.083	-5.531
*ilmamaa*	world (poetic)	2.344	6.916	7.825	-5.481
*ideaalmaastik*	ideal landscape	2.188	6.529	7.600	-5.412
*surmatants*	dance of death	2.505	6.946	7.843	-5.338
*tähesõda*	star war	2.205	6.209	7.414	-5.209
*sfäär*	sphere	2.686	6.921	7.828	-5.142
*pallivõlur*	ball wizard	2.690	6.905	7.819	-5.129
*kevadring*	spring circle	1.931	5.558	7.034	-5.104

The column Predicted shows the predicted value of the rating in the present dataset based on the Aedmaa et al. (2018) dataset. The column Residual shows the model residuals, i.e., the difference between the observed and predicted values

**Table 2:** Top ten words with largest positive model residuals, i.e., words which were rated to be significantly more concrete in the present study than in Aedmaa et al. (2018)

word	Translation	Proos & Aigro	Aedmaa	Predicted	Residual
*püü*	lagopus	9.643	1.125	4.453	5.190
*eeskiri*	rule	9.402	1.794	4.843	4.559
*miinusmärk*	minus sign	9.281	1.786	4.838	4.443
*pandipidaja*	pledgee	9.285	1.881	4.893	4.392
*tollimaks*	duty tax	9.580	2.578	5.299	4.281
*üleeilne*	from the day before yesterday	9.569	2.600	5.312	4.257
*laenuvõtmine*	loan-taking	9.324	2.230	5.097	4.227
*öeldis*	verb	8.869	1.508	4.676	4.193
*juunikuu*	june	9.355	2.381	5.185	4.170
*küsimärk*	question mark	9.029	1.827	4.862	4.167

The column Predicted shows the predicted value of the rating in the present dataset based on the Aedmaa et al. (2018) dataset. The column Residual shows the model residuals, i.e., the difference between the observed and predicted values

Table 2 on the other hand illustrates a range of words judged as more concrete by humans than by machine learning algorithms. One set that emerges in this list is made up of bureaucratic or formal concepts, the context of which is likely to be made up of highly abstract lexemes, but which nevertheless affect people (raters) directly and strongly (*laenuvõtmine* ‘loan-taking’, *eeskiri* ‘rule’). Concepts related to such experiences as writing and orthography (*miinusmärk* ‘minus sign’ and *jutumärk* ‘quotation mark’) may also be susceptible to this effect, with machine learning algorithms not picking up on the strongly physical aspect of the act of writing.

### Semantic ambiguity

In large human rating studies, semantic variation is not usually addressed, and in some cases, words with multiple meanings are even excluded (Lahl et al., 2009). This has been criticized by Gilhooly and Logie (1980) and Reijnierse, Burgers, Bolognesi, and Krennmayr (2019) among others. As stated in “Material” section, for the homonyms among our stimuli, we included their distinct meanings as separate stimuli. Ratings reveal that the difference between ratings of distinct meanings with identical form varies greatly. Table 3 includes two homonyms with very different ratings (top four rows), and two homonyms with very similar ratings^4^ (bottom four rows).

As the present ratings only explore one semantic facet of the concepts — concreteness — it is to be expected that homonyms representing different concepts are nonetheless rated more or less same in terms of their concreteness (e.g., *palk* ‘wage’ and *palk* ‘log’), i.e., conceptually very different words can still have the same level of concreteness. For words that do represent concepts that differ from each other drastically in terms of their concreteness, making this distinction important in order for them to be usable in any other studies, as exemplified by *arm* ‘scar’ and *arm* ‘love’ in Table 3.

## Discussion

This paper presented a dataset of concreteness ratings for 35,979 Estonian words, collected in a first ever human rating task in this language. Similarly to the dataset of just more than 2,000 words in Lahl et al. (2009), our ratings reflect a preference for the concrete side of the scale, rather than the abstract side (low concentration of ratings in the 0–4 range, and high concentration in the 5–9 range). Participants were more likely to agree on which words are highly concrete than on highly abstract or semi-concrete words. This is not an unusual finding as the middle of the rating scale has been noted to have a higher rate of disagreement (Pollock, 2018; Neath & Surprenant, 2020). Pollock (2018) has analyzed the norms from Brysbaert et al. (2014b), and argues that the middle of the rating scale seems to not be indicative of the concreteness or abstractness of the concepts, but rather of the participants disagreeing on the rating. Thus, concepts from the middle of the scale should always be used carefully in further studies, and notice has to be made in case of large SDs. Neath and Surprenant (2020) agree with Pollock (2018) in this aspect and argue that better results are achieved by controlling for multiple variables at once, e.g., effectiveness and frequency should be controlled for in addition to concreteness. Nevertheless, Neath and Surprenant (2020) also show that large SDs did not have any effect on the explanatory power of concreteness ratings and as such, a large SD does not indicate that the rating is unfit for use.

As stated in “Overview of collected ratings” section, we did not exclude any ratings on the basis of their standard deviation. While we do agree that factoring in SD is crucial when using any kind of ratings, we agree with Neath and Surprenant (2020) in that controlling for multiple variables at once is the best course of action. Furthermore, although caution is advised when considering ratings with high SDs as a variable, the information about the words themselves is still useful. High disagreement rates can tell us something about the word itself as well, for example, it might be polysemous or the concept it rates might be highly dependent on personal experiences.

The present paper highlighted the role of collection method in concreteness ratings. In our comparison of machine and human ratings, we report slightly lower overall correlation than previous studies: 0.71. The two datasets were shown to differ both in terms of the shape of distribution (unimodal for human raters, bimodal for machine learning), as well as in terms of the overall concreteness of ratings. Machine-based ratings were generally more abstract, with a high peak in the in the 1–2 rating region. Humans, however, were more likely to rate words as concrete. It is important to point out that Aedmaa et al. (2018) took machine translation equivalents of Brysbaert et al. (2014b) as the training data for Estonian indexes. Hence, the algorithm was based on human judgements but not on those originating from Estonian speakers. This could account for some of the discrepancy as two seemingly semantically equivalent words might be pragmatically very different, which could amount to a misrepresentation of the concreteness level, which in turn would have a carry-over effect to the subsequent machine ratings.

**Table 3:** Homonyms with very different ratings (top four rows) and very similar ratings (bottom four rows)

Homonym	Translation	Rating
*arm...armi..armi*	scar	7.926
*arm...armu..armu*	love	3.62
*kile...kile..kilet*	film/membrane	8.197
*kile...kileda..kiledat*	shrill	2.326
*palk...palga..palka*	wage	8.715
*palk...palgi..palki*	log	9.214
*mark...marga..marka*	mark (currency)	7.748
*mark...margi..marki*	stamp	7.837

However, at least some degree of the distinctions found between the two datasets likely originates from context availability, and the semantic and morphological composition of the words. As discussed in “Results” section, the concreteness of compounds seems to differ between two collection methods (human and machine), most likely due to the differences in context availability for human vs. machine raters. Although neither type of ratings is context-free, the degree and nature of the context is different. For human raters, assigning a rating to a word requires the participant to imagine at least some type of context for it. Furthermore, a concept inherently includes the variety of contexts in which the speaker has encountered the concept. However, in a task without explicit context, human raters might have trouble assigning specific semantic content to the word. A machine rater, on the other hand, bases its decisions on the explicit context it encounters the word in – if the context includes highly concrete words, the word under question will also receive a highly concrete rating.

Another aspect that the algorithm does not take into account is that a word’s overall concreteness can be based on the intensity of personal experience a speaker has with the concept. That is, items with no physical bodies or available imagery may nevertheless induce intense experiences, leading human raters to assess them as concrete (e.g., *eeskiri* ‘rule’/‘regulation’). An algorithm cannot take this dimension of concreteness into consideration.

Finally, one can assume that the way polysemy and homonymy are handled by the human raters versus machine rater might have an effect on the outcome. We showed in “Semantic ambiguity” section how specifying the meaning of homonyms resulted in vastly different ratings for some of the words. However, we also showed that for some of the words, the differences in ratings was negligible. In the case of automatic rating assignment, semantically ambiguous words with very different meanings likely get an ‘evened out’ score. However, it is important to note that in the case of polysemy, for example, we also do not know how the human rater interprets the meaning. If different raters interpret separate meanings, the same ‘evening out’ pattern would be true for human ratings as well. Thus, semantic ambiguity remains a challenge for both types of ratings.

Our database differs from most others in that we provide differentiated ratings for the homonyms in our dataset. Words with multiple meanings are prevalent in all languages, and it would benefit researchers if semantic variation would be taken into account in rating studies. We do acknowledge, however, that as Reijnierse et al. (2019) point out, polysemy is as important as homonymity when considering semantic variation. However, looking at how frequent polysemy is in languages, and how difficult it is to pinpoint the number of polysemous meanings a word has, let alone describe these different senses meaningfully and unambiguously to participants, this is not feasible for a large-scale study. Thus, the caveat persists with the present data that polysemous words are not differentiated, and this should be taken into account when using the ratings.

Another facet that our study addresses is the choice of scale. Instead of using the 1–7 Likert scale that has been utilized in most concreteness rating collections, we used a 0–10 continuous scale, where participants had the opportunity to assign values with decimal points. There are several upsides to this approach. First, one obtains a much more detailed dataset with regard to concreteness indexes. Second, the data are more suitable for various statistic metrics. Liddell and Kruschke (2018) and Taylor, Rousselet, Scheepers, and Sereno (2022) discuss the issue that studies frequently use metrics on semantic and psychological ratings which are designed for numeric rather than ordinal data. The present method bypasses this issue by using a continuous scale.

Considering the added bonus of allowing participants to assign more specific ratings, increased reliability of some common statistical metrics, indications that continuous scales offer ratings which are as reliable as ordinal scales (Albaum, Best, & Hawkins, 1981; Imbault, Shore, & Kuperman, 2018), and the ease of using a continuous scale offered by current technological solutions, we might expect to see more continuous scales used in the future. It would also be beneficial to conduct comparative studies between the two types of scales, as the current research on the topic is limited.

The method does come with its own set of drawbacks as well. For example, it is difficult to exclude participants based on their rating patterns. One of the criteria we used to exclude participants was giving *exactly* the same ratings to at least 30 consecutive items: 46 participants were excluded based on this criteria. This means that they clicked on the same number underneath the scale 30 times in a row, as one could click on numbers as well as drag the indicator to establish a rating. However, this does not address the participants who simply dragged the indicator to a random place on the scale, or to a highly similar place. We adapted this criterion from previous studies (Xu & Li, 2020; Soares et al., 2017; Lahl et al., 2009) with an increased threshold, i.e., we used a lower number of consecutive ratings as our cut-off point. Thirty items would amount to $\tilde{1}$5% of the total words rated, which we hoped to be strict enough. Still, this method could not be sensitive enough to detect non-compliance. We do hope, however, that by applying a number of different criteria for excluding participants, we were able to manage the risks brought on by the nature of a continuous scale.

Finally, while rating scales still remain the most applied method for collecting semantic ratings, other methods are also gaining popularity. For example, best-worst scaling is one method that has been utilized. Hollis and Westbury (2018) show that best-worst scaling offers scores with better predictive power than rating scales for the semantic characteristics of age of acquisition, arousal, and concreteness. Crucially, the method offers better results with less observations per word (Kiritchenko & Mohammad, 2017; Hollis & Westbury, 2018), making it less resource-demanding than the traditional rating scales. De Bruyne, De Clercq, and Hoste (2021) show that out of rating scales, pairwise comparison and best-worst scaling, the latter had the highest participant agreement rate, which also speaks to the higher reliability of best-worst scaling. Thus, in the future, it would be beneficial to apply this method for collecting semantic norms, especially in the case of small populations, such as Estonian native speakers.

## Data Availability

The datasets generated and analyzed during the current study, and the analysis scripts are available in the OSF repository, LINK TO REPOSITORY.

## Appendix. Instructions in Estonian

Meie katse uurib sõnade konkreetsust ja abstraktsust. Mõned sõnad on konkreetsed, sest nad on väga selgelt viie meelega (nägemine, kuulmine, haistmine, maitsmine, kompamine) tajutavad - näiteks “pastakas”. Teised sõnad on aga abstraktsed, sest neid on raske meeltega sellisel moel tajuda, kuid nad on sellegipoolest olemas - näiteks “sõprus”. Hulk sõnu langeb aga nende kahe äärmuse vahele, sest ühelt poolt on neid kohati võimalik meeltega tajuda, teiselt poolt toetume nende mõistmiseks aga ikkagi keelele.

Selliseid hinnanguid nimetatakse sõna konkreetsushinnanguteks. Konkreetsushinnangud on väga väärtuslik teadusressurss, mida saab kasutada nii keeleteaduses, psühholoogias, keeletehnoloogias, aga ka näiteks turunduses. Selle uuringu tulemusena valmib konkreetsushinnangute andmebaas, mis on vabalt ligipääsetav kõigile.

Hakkad ükshaaval nägema eestikeelseid sõnu. Palume sul iga sõna juures hinnata, kui abstraktne või konkreetne on see sõna skaalal nullist kümneni, kus null on kõige abstraktsem ja kümme kõige konkreetsem. Võid hinnangut andes valida ka väärtuse täisarvude vahel, näiteks 4,6. Enne, kui katse algab, saad mõne sõna peal ka harjutada.

Katses näed sõnu erinevates vormides (nt “parkiv”, “ilusam”, “puu”, “tegemas”). Anna oma hinnang sõnale täpselt selles vormis nagu seda näed. Kõik nimisõnad on ainult ainsuses ja nimetavas käändes. Näiteks sõna “laud” viitab mööbliesemele mitte silmalaugudele, “looma” viitab loomise tegevusele, mitte sõna “loom” omastavale või osastavale. Kõik tegusõnad on -ma või -da vormis. Seega ei kohta sa sõnu nagu “mängin” või “kadusime”.

Kui näed sõna, mille tähendust sa ei tea, siis pane hinnangu asemel linnuke skaala all olevasse kasti. See võib väga lihtsalt juhtuda, sest kokku on meie nimekirjas 40 000 erinevat sõna (millest sina näed 215) ning seda on palju rohkem, kui keskmise inimese sõnavara. Vahel võib ka juhtuda, et mõni sõna ilmub sulle katse jooksul mitu korda.

## Footnotes

1 This number was confirmed based on reference dictionary EKSS (Langemets, Tiits, Valdre, Veskis, Viks, & Voll, 2009)

2 Participants saw all instructions in Estonian. For ease of reading, we only include the shortened English translation here. *Verbatim* full instructions are provided in the Appendix.

3 We used the *cocor* package (Diedenhofen & Musch, 2015) for the calculation.

4 The full list on homonyms with respective ratings is provided in additional materials in the OSF repository
